# Inflammatory myofibroblastic tumor in the liver after bone marrow transplantation: case report and literature review

**DOI:** 10.3389/fmed.2025.1489399

**Published:** 2025-03-24

**Authors:** Shuhui Yang, Yongsheng Tang, Zenan Yuan, Jianwen Zhang

**Affiliations:** Department of Hepatic Surgery and Liver Transplantation Center, Third Affiliated Hospital of Sun Yat-sen University, Guangzhou, China

**Keywords:** anaplastic lymphoma kinase (ALK), case report, immunohistochemistry, inflammatory myofibroblastic tumor, liver tumor, MRI

## Abstract

**Introduction:**

Inflammatory myofibroblastic tumor (IMT) is a rare low-grade malignant neoplasm in the liver. Timely diagnosis and treatment of IMT are challenging due to its atypical symptoms and imaging results.

**Case report:**

We report a 46-year-old woman who presented to our hospital with persistent hyperpyrexia and discomfort in the right upper abdomen for 2 months post bone marrow transplantation. Radiological findings revealed a space-occupying lesion of uncertain nature in the liver. Since the histological examination of the biopsy specimen indicated IMT, she underwent surgical resection. Subsequently, the postoperative pathology confirmed the diagnosis of IMT. The patient’s febrile condition subsided after the surgery. A magnetic resonance imaging (MRI) scan performed 8 months later showed no signs of recurrence.

**Conclusion:**

IMTs are caused by genetic rearrangements. Diagnosing IMT can be challenging especially in this case as we had to differentiate the tumor from inflammatory diseases associated with bone marrow transplantation. Hence, a thorough pathological immunohistochemical examination is required to confirm its diagnosis. Local IMTs should be treated with radical surgical resection. In cases of distant metastasis or incomplete resection cases, chemotherapy, targeted therapy, or immunotherapy can be utilized. Regular follow-up is crucial for improving the patient’s survival rate.

## Introduction

Inflammatory myofibroblastic tumor (IMT) is a rare neoplasm that originates from mesenchymal cells (1). It is mostly observed in the lungs, greater omentum, and mesentery. Although rare, it may also occur in other organs (2). Previously known as inflammatory pseudotumor (IPT) because of its histological features of spindle cell proliferation and inflammatory cell infiltration (3), IMT is now considered a neoplastic lesion rather than a simple inflammatory reaction (1, 4). The occurrence of this condition in the liver is quite rare. Its atypical symptoms and radiographic manifestations complicate the diagnosis and can lead to confusion with other liver tumors. Therefore, immunohistochemistry is essential for diagnosing IMT. In this report, we describe the clinical and imaging features, treatment methods, and prognosis of a patient at our medical center diagnosed with IMT following bone marrow transplantation. The patient’s prognosis improved after surgical resection. This case report offers reliable and practical guidance for diagnosing hepatic IMT and highlights the significance of surgical treatment in these cases.

## Case presentation

A 46-year-old woman was admitted to our hospital with fever, mild discomfort in the right upper abdomen, and intermittent shoulder as well as lower extremity myalgia for last 2 months. She did not complain of pharyngalgia, cough, or diarrhea. An abdominal computed tomography (CT) scan performed 1 month back in a nearby hospital had revealed an abnormal density lesion in the S6/7 segments of the liver. Leukemia recurrence was suspected due to her hospitalization for acute myeloid leukemia (AML) and her bone marrow transplant 6 months earlier. Consequently, she underwent bone marrow and percutaneous liver biopsies. The results of these biopsies indicated complete remission of AML. The pathological findings of liver biopsy revealed inflammatory granulation tissues. Subsequently, an infectious etiology was suspected, and anti-infective therapy was initiated. Since her body temperature remained significantly elevated (up to 40°C), she was referred to our hospital. At admission, she weighed 42 kg, was 160 cm tall, and had a body mass index (BMI) of 16.4. She continued taking cyclosporine tablets daily. Additionally, she had hypoproteinemia, bilateral pulmonary scattered inflammatory infiltrates, and minor pericardial effusion. She had an unremarkable psychosocial and family history and denied any previous other chronic diseases.

Her physical examination demonstrated that her abdomen was flat and pliable with no palpable tenderness.

Her laboratory investigations revealed enhanced white blood cell count (17.75/mL), significant iron deficiency anemia with a hemoglobin level of 57 g/dL, a platelet count of 459/mL, and a procalcitonin level of 0.15 ng/mL. The prothrombin time was marginally extended to 15.8 s. Tests for hepatitis B virus DNA and 2019 Novel Coronavirus (19-nCoV) nucleic acid assay results were negative. Her biochemical laboratory parameters were normal, except for a significantly elevated serum ferritin level of 2,140 ng/mL (normal range: 10–291 ng/mL).

**Figure 1:**
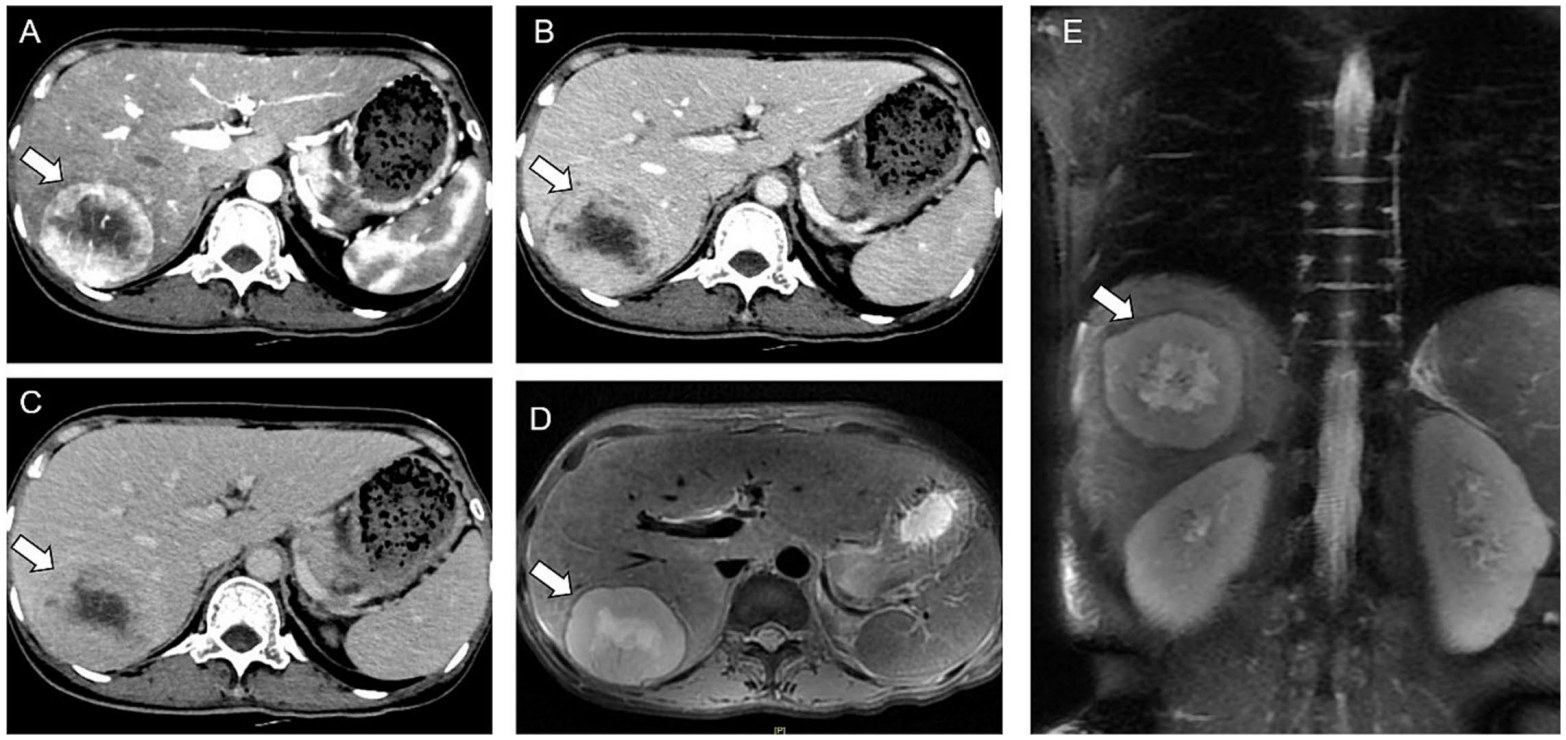
Computed tomography (CT) and magnetic resonance imaging (MRI) results. **(A–C)** Arterial, venous, and delayed phases of enhanced CT. **(D,E)** MRI shows a huge round mass.

A CT scan identified a hypodense tumor, measuring 67 × 58 mm, with an indistinct margin, in the S6/7 segments of the liver. The arterial phase showed the tumor’s heterogeneous enhancement, while the portal venous and delayed phases revealed a reduced enhancement. The tumor had a visible capsule encircling a necrotic core that failed show enhancement (Figures 1A–C). Additionally, the MRI depicted the mass as hypo-intense on T1-weighted images and hyperintense on T2-weighted images. The arterial phase showed a heterogeneous enhancement, while the venous and delayed phases revealed progressive and slightly diminished enhancements (Figures 1D,E).

**Figure 2:**
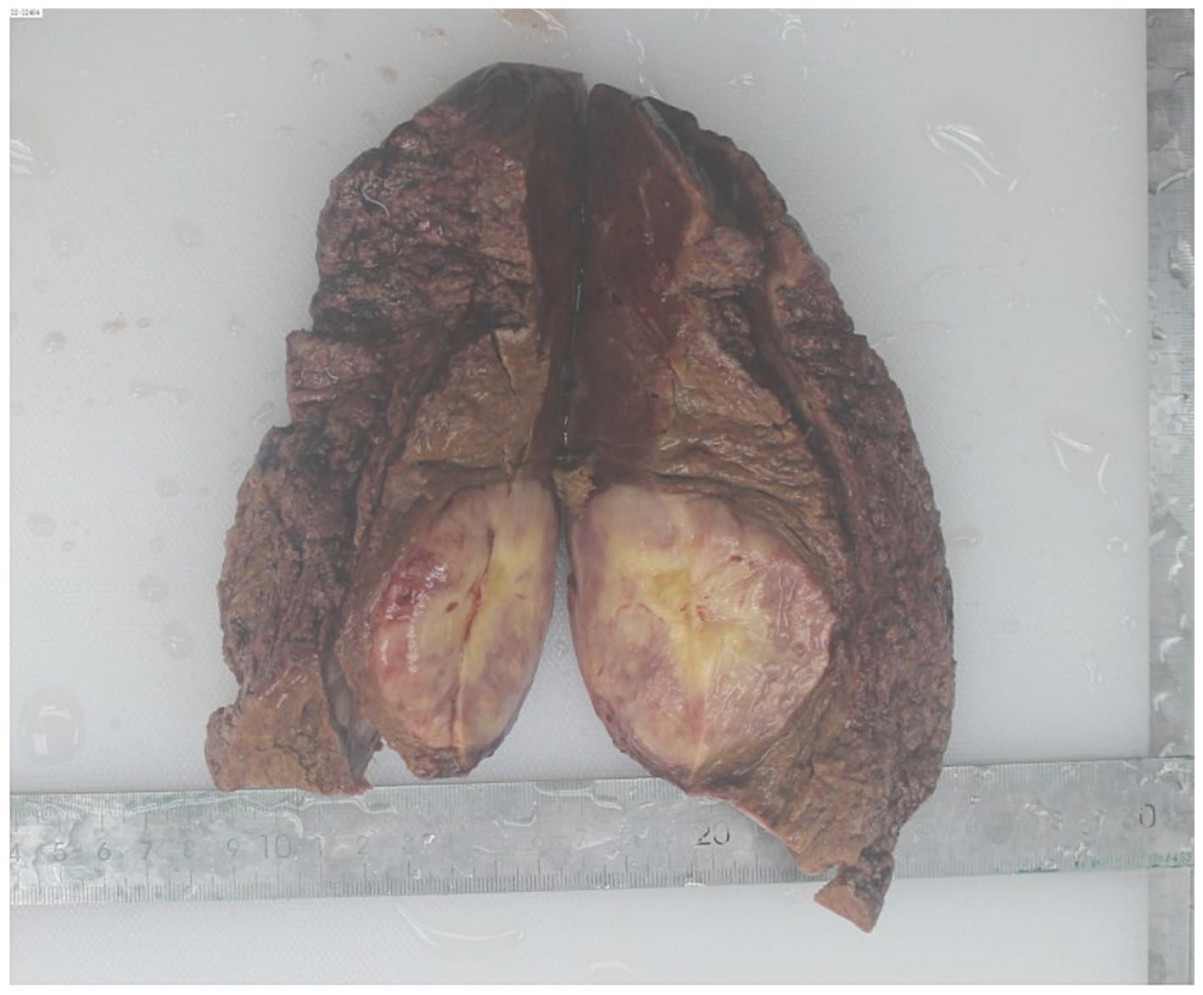
Macroscopic pathology of inflammatory myofibroblastic tumor (IMT) postoperatively.

**Figure 3:**
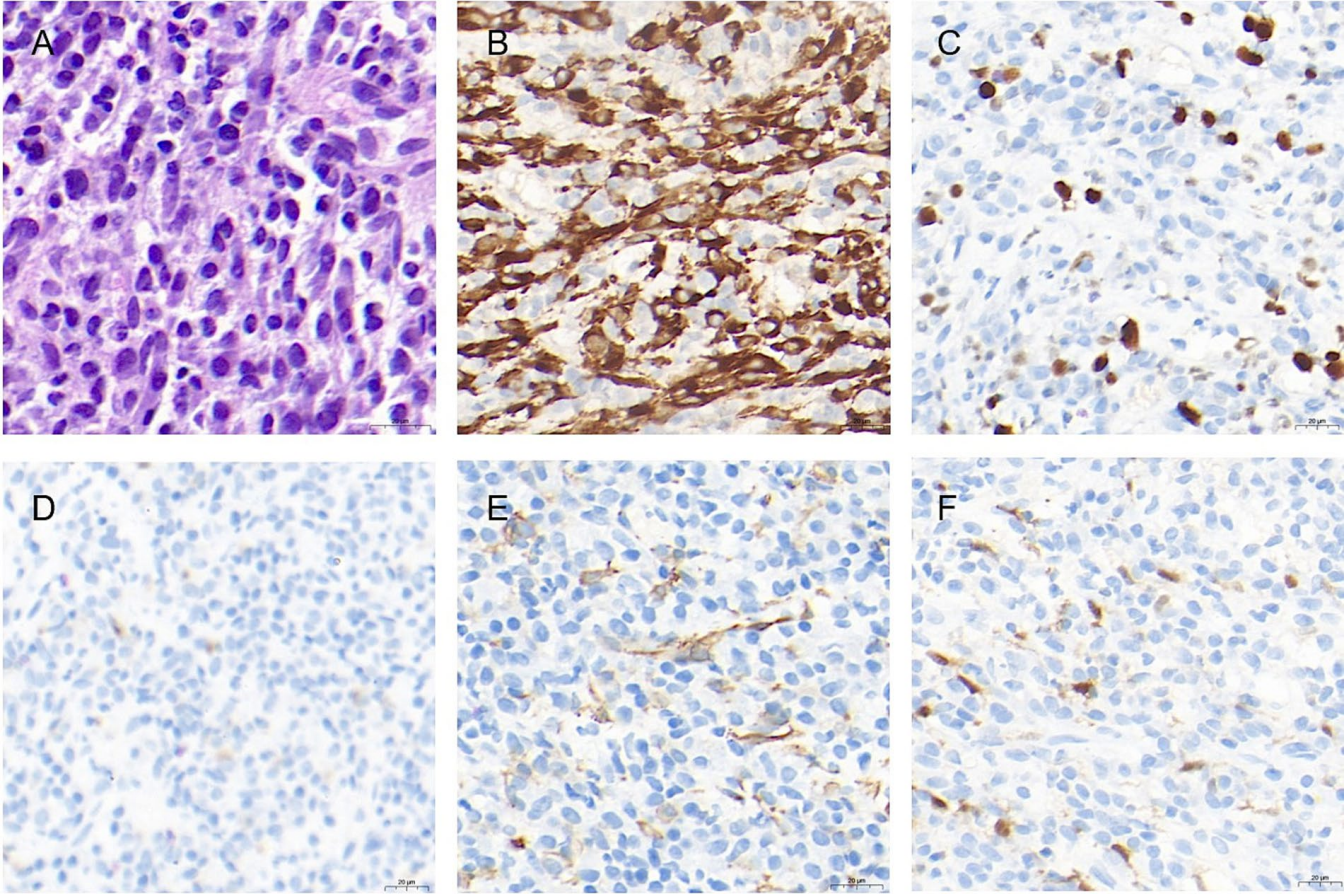
Staining of the tumor specimen. **(A)** Proliferation of spindle-shaped cells, with heterogeneity, enriched nucleoli, and mitotic figures. Multiple lymphocytes, plasma cells, and neutrophils were seen infiltrating the stroma (hematoxylin and eosin stain, ×70). **(B)** Immunopositivity for anaplastic lymphoma kinase (original magnification ×50). **(C)** Approximately 10–15% of Ki-67 immunopositivity (original magnification ×50); **(D)** Desmin immunopositivity (original magnification ×50). **(E)** Immunopositivity of smooth muscle actin (original magnification ×60). **(F)** S100 immunopositivity (original magnification×50).

Given the patient’s history of leukemia and lack of any previous episodes of hepatocellular carcinoma related to hepatitis or other etiological factors, the probability of leukemic hepatic infiltration was considered. A percutaneous liver tumor biopsy was performed. The pathological results of the tumor biopsy identified it as an inflammatory fibroblastic tumor. Subsequently, the patient underwent a laparoscopic anatomic resection of the right posterior hepatic lobe. Intraoperatively, the liver exhibited a soft parenchymal texture, and certain areas exhibited a bluish discoloration, particularly in the right posterior hepatic lobe. This was attributed to hepatic sinusoidal stasis post chemotherapy. The tumor measured approximately 2.6 × 5 × 5 cm and was firmly adherent to the diaphragm. Considering the tumor’s invasion of the diaphragm, an open surgerical approach was adopted. The tumor, along with a segment of the diaphragm, was excised en bloc; the diaphragm was then repaired by continuous suturing with a barbed wire, and an indwelling thoracic drainage catheter was placed. The surgery lasted for 5 h and 50 min, with a total blood loss of 500 mL. Postoperatively, an immunohistochemical staining assay identified the gross specimen as an IMT. The tumor surface exhibited a grayish-yellow hue with a distinct margin, and the cut surface was grayish-yellow intermingled with red (Figure 2). The histopathological evaluation revealed that the tumor comprised spindle cells arranged in a scattered or fascicular pattern. The heterogeneous cells with nucleoli and mitotic figures revealed focal necrosis. Numerous lymphocytes, plasma cells, and neutrophils were infiltrated within the stroma. Immunohistochemical staining results revealed positive outcomes for smooth muscle actin (SMA), anaplastic lymphoma kinase (ALK), S100, desmin, and myeloperoxidase (MPO). However, the Ki-67 index exhibited approximately 10% positivity (Figure 3). Additionally, hepatitis-associated antigen (HEP) and Epstein–Barr virus were not detected.

**Figure 4:**
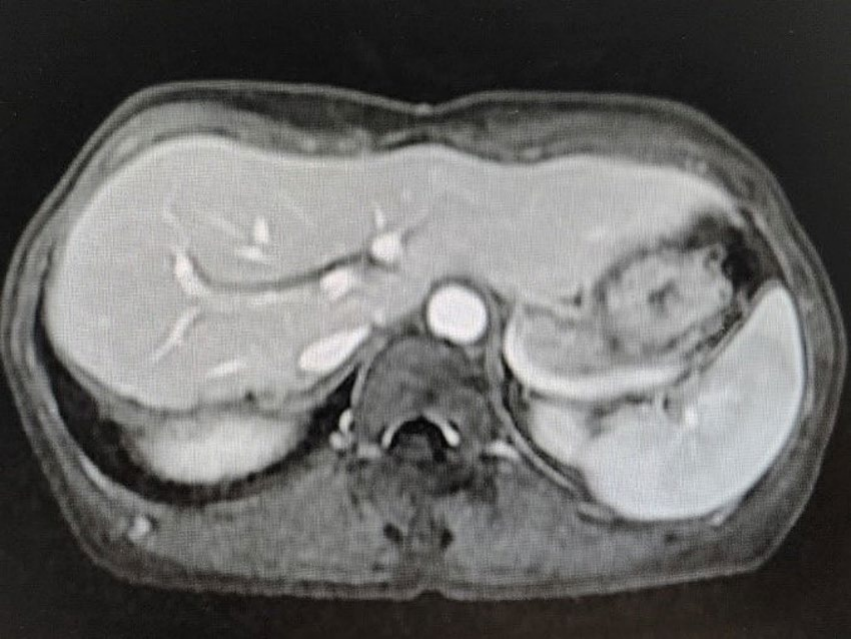
A postoperative MRI scan 8 months later showed no evidence of IMT recurrence.

The patient’s febrile episodes ceased postoperatively, and no additional postoperative complications were observed. She was discharged on the ninth postoperative day. The patient demonstrated good compliance and attended regular outpatient follow-up appointments without any adverse events (Table 1). A postoperative MRI scan after 8 months showed no evidence of recurrence (Figure 4).

**Table 1:** Timeline.

August 2021	Diagnosed with acute myeloid leukemia
February 2022	Underwent bone marrow transplantation
June 2022	Initial symptoms: fever with mild discomfort in the right upper abdomen and intermittent myalgia in the lower extremities and shoulders
6 June 2022	Abdominal CT: abnormal density lesion in the s6/7 segments of the liver Bone marrow biopsy: complete remission of AML Percutaneous liver biopsy: inflammatory granulation tissue
June to August 2022	Anti-infective therapy
17 August 2022	Hospitalization
19 August 2022	Abdominal CT: a hypodense mass in the s6/7 segments of the liver, which was considered to be hepatocellular carcinoma MRI of the abdomen: the s6/7 segments of the liver are occupied; leukemic hepatic infiltration was not excluded
26 August 2022	Tumor biopsy: inflammatory fibroblastic tumor
28 August 2022	Discussion with the surgery team; surgical treatment was elected
29 August 2022	Surgical treatment
7 September 2022	Discharge from hospital
April 2023	Latest follow-up

We inquired about the patient’s opinions regarding the treatment 8 months postoperatively. She expressed her satisfaction with the course of the treatment, noting that the treatment alleviated her high-grade fever, which had been severely impacting her daily activities and quality of life. She appreciated the speedy postoperative recovery and readily agreed to the publication of her case as she considered that it might benefit other IMT patients.

## Discussion

IMT is a rare mesenchymal neoplasm that is currently classified as an intermediate-grade malignancy. Characterized by low-grade malignant features, it has a lower metastatic rate but a higher risk of recurrence (5). IMT is more common in children and young adults, with a higher prevalence in women than in men (6). The etiological underpinnings are unknown. A previous study has suggested its potential association with viral infections, immunosuppressive conditions, and traumatic injuries (7). However, this perspective might be attributed to a limited understanding of IMT in previous studies because they did not specifically elaborate the mechanism. Parker et al. (4) suggested that the laboratory analysis and the inflammation-associated manifestations are its outcomes rather than etiological factors. In light of recent developments, IMT can be now regarded as a neoplastic transformation; however, its local invasiveness also possesses the characteristics of a malignant tumor (6, 7). Gene rearrangements are the probable causes of tumorigenesis. Approximately 50% of IMT cases possess ALK gene rearrangement, which causes IMT to be ALK-positive (8). The ALK gene encodes tyrosine kinase receptor, which was initially discovered in mesenchymal large cell lymphoma and is usually expressed only in neural tissues (9). Therefore, ALK positivity can be used for distinguishing between IMT and inflammatory pseudotumors. Although many patterns and fusion proteins leading to ALK gene fusion have been identified, the staining pattern of ALK is linked to fusion proteins (6). However, the molecular configuration of ALK gene fusion partners in significant IMT cases comprises transcriptional activator elements and oligomerization motifs, which causes aberrant activation of ALK (7, 10–13). Currently, available cases have not been evaluated regarding the types of gene fusion and prognosis. However, another study has reported that ALK positivity might indicate a good prognosis (6), while patients with gene rearrangements involving both RAN binding protein 2 (RanBP2) and ALK exhibit a higher tumor recurrence rate and a relatively poor prognosis (14).

Histologically, IMT possesses different morphological features ranging from inflammatory pseudotumor to malignant sarcoma, associated with various gene rearrangements. Some cases have a typical morphology. The study by Antonescu et al. (6) reported that in ALK rearranged cases, tumors comprised mild spindle cells, with low mitotic activity, mild-to-moderate inflammation, and fibromyxoid stroma. However, the tumor cells exhibited round cell morphology in RanBP2 and ALK gene rearrangements cases (14–16). Immunohistochemistry can be useful for investigating the nature and origin of the tumor in IMT cases. In liver IMT cases, it is important to distinguish the IMT from the gastrointestinal stromal tumor (GIST) and liver metastasis. The presence of abundant myofibroblasts in IMT cases can help in confirming the diagnosis. However, CD117 positivity is observed in GISTs (17), which is different from IMTs. Several muscle cell markers such as vimentin, SMA, and desmin can be used to identify the tumor originating from mesenchymal tissues as an auxiliary diagnosis (18). Owing to the scarce prevalence of IMT, the diagnosis of ALK-negative IMT cases becomes challenging. Thus, the diagnostic modality of IMT remains exclusive and is primarily based on pathological findings (19).

Clinically, IMT exhibits a few inflammation-related symptoms, such as pyrexia, emaciation, and abdominalgia. Laboratory tests reveal common findings such as microcytic hypochromic anemia, reactive thrombocytosis, and elevated erythrocyte sedimentation rate (ESR). Although these findings are similar to those of malignant tumors, they lack specificity in the context of IMT diagnosis. Moreover, a delay in diagnosis and treatment might worsen the prognosis. The liver is a rare site for IMT, accounting for only a few cases (Table 2). Moreover, abdominal pain and fever are more prevalent in patients with hepatic IMT, compared to those with IMTs occurring in other organs (20). Since IMT commonly comprises inflammatory and fibrotic constituents, it may exhibit moderate to significant homogeneous or heterogeneous enhancements in contrast-enhanced scans (21). In the study by Gros et al. (22), imaging often shows low-density shadows on CT scans. Additionally, MRI might not show specific diffusion-limiting features (23). Moreover, the clinical manifestations of IMT are significantly associated with its location (6).

**Table 2:** Case report.

No.	References	Age/gender	Symptoms	Radiological findings	Immunohistochemistry (positive result)	Lab results	History	Treatment	Follow-up
1	Fangusaro et al. (31)	10 years/male	Abdomen distended	A round solid mass of 7 cm in the inferior portion of the right lobe of the liver CT: Hypervascular with central diffuse enhancement and several faint calcifications	SMA, ALK	NM	ALL, bone marrow transplantation	Hepatectomy	No recurrence 7 months after surgery
2	Lorenzi et al. (12)	24 years/male	General discomfort, jaundice	A solid mass of 4 cm in diameter involving the hepatic hilus	SMA, desmin, ALK	Elevated transaminases	NM	Crizotinib (250 mg twice daily)	Disease was stable for 4 months
3	Dasgupta et al. (32)	7 years/male	Afebrile, jaundice, tender hepatomegaly	A 5-cm tumor mass at the hilum of the liver extending into segments 4 and 8 and along the right and left divisions of the hilar structures CT: delayed enhancement	Actin, vimentin	Mild anemia, alkaline phosphatase of 3,855 IU/L, prothrombin time of 16.3 s, fibrinogen concentration of 5 g/L, CRP at 86 mg/L, IgA at 3.86 g/L, IgM at 2.81 g/L	Vomiting, diarrhea	Hepatic transplantation	No recurrence 15 months later
4	Solomon et al. (33)	26 years/male	Pain in the right upper quadrant, weight loss, fatigue, hepatomegaly	2 contiguous, infiltrating hepatic masses measuring 6.7 × 5.5 × 4.4 cm and 6.2 × 6.0 × 3.9 cm, respectively	Vimentin, SMA	Normochromic anemia, elevated ESR	NM	Hepatectomy	NM
5	Chen et al. (14)	34 years/male	Abdominal fullness in the right upper quadrant, weight loss	A hypervascular hepatic tumor at segments 5 and 6 with suspicious extrahepatic extension	Vimentin	Mild leukocytosis (12,190 cells/μL)	NM	Hepatectomy	Recurred 5 months later and the patient died
6	Sürer et al. (34)	48 years/female	Weakness, fever, weight loss, pain in the right upper abdomen, the right upper quadrant was tender at palpation	A single hypoechoic lesion measuring 6 cm in greatest diameter in the right lobe of the liver	SMA, ALK	WBC count of 10,900/μL, ESR of 120 mm/h, Hb level of 7.7 g/dL	NM	Hepatectomy	No recurrence 2 years later
7	Manolaki et al (35)	9 years/female	Fever, mild anorexia, weight loss, intermittent epigastric pain	Liver ultrasound (US) revealed a hypoechoic lesion (3.5 × 2.5 cm) CT: a hypodense space-occupying lesion	Vimentin, SMA	WBC count of 15,600/mm^3^ and ESR of 97 mm	*Mycobacterium tuberculosis*	Hepatectomy, a 9-month course of isoniazid and rifampicin in combination with pyrazinamide for the first 2 months	No recurrence 3 years later
8	Kruth et al. (13)	41 years/female	Fever (40°C)	MRI: a liver tumor of 3.3 cm in diameter in segment 6	Actin	Elevated transferase, leucocytes of 16.369/uL, CRP of 263 mg/L, pro-calcitonin of 1.54 μg/L microalbuminuria of 602 mg/L (0–20), and proteinuria of 935 mg/L (0–150)	NM	Hepatectomy	No recurrence 1 year later
9	Chablé-Montero et al. (36)	23 years/female	Fever, diaphoresis, pain in the right hypochondrium	CT: a heterogeneous rounded hepatic lesion of 7 cm in greatest dimension	SMA	Leukocytosis	NM	Hepatectomy	NM
10	Nagarajan et al. (29)	15 months/NM	Enlarged liver	Abdominal sonogram showed a 4.6 cm × 4.6 cm × 5.0 cm mass in the left lobe of the liver with central calcification. CT: enhancement of the mass on the portal venous phase	NM	Hb level decreased, platelet count of 868,000 cells/μL, serum iron of 14 mg/dL, total iron-binding capacity (TIBC) of 252 mg/dL, transferrin saturation of 6%, serum ferritin of 140. CRP of 11.9 mg/L, ESR of 70 mm/h	NM	Hepatectomy	No recurrence 1 year later
11	Kim et al. (37)	5 months/male	Enlarged liver	Ultrasound scan showed about 10 cm-sized mass in the right lobe of the liver MRI: A 10.4 × 9.5 × 9.0 cm mass, low signal intensity on T1-weighted images, heterogeneous signal intensity on T2-weighted images, positive enhancement, and diffusion restriction on diffusion-weighted images	SMA, ALK	NM	NM	Hepatectomy	No recurrence 14 months later
12	Sinha et al. (38)	36 years/female	Bloating of the abdomen, dyspepsia	GB shows mild diffuse wall thickening with asymmetric wall thickening in the body region extending along the adjacent duodenum with circumferential thickening of the duodenal wall causing slight luminal narrowing with mild proximal gastric distention. Fat plane between GB and lesion poorly visualized with mild hepatic altered attenuation (depth up to 1 cm)	NM	NM	NM	Extended cholecystectomy + Billroth II gastrectomy	NM
13	Shang et al. (39)	54 years/female	Tarry stools	CEUS examination: enhancement in the arterial phase, quickly faded, hypo-enhancement in the portal venous and late phases CT: a low-density mass	SMA, vimentin, actin, CD34	Decreased red blood cell, WBC, platelet, Hb, and albumin levels Elevated transferase, prothrombin time, d-dimer, blood urea nitrogen, and creatinine levels	HBV, liver transplant	Conservative therapy: antibiotics, steroids, antiviral agents, tumor necrosis factor-blocker, biliary drainage, and endoscopic ligation of esophageal varicosity	Patient died 2 months later
14	Watanabe et al. (40)	70 years/female	NM	CT: unenhanced and low-density, heterogeneous and increasing mass	SMA, cytokeratins, CK18	Elevated CRP levels	Lung cryptococcosis, traffic accident	Hepatectomy	No recurrence 7 months later
15	Filips et al. (41)	32 years/female	Fever, anemia, malaise, pain in the right flank	Ultrasound: a round, encapsulated liver lesion PET CT: a metabolically active tumor, an 8 cm × 8 cm tumor	SMA, ALK	Transferase increased	Postpartum	Hepatectomy	No recurrence 1 year later
16	Li et al. (42)	29 years/male	Weakness, slight discomfort in the upper abdomen	Ultrasound: a low-echo area of 67 mm × 64 mm MRI: T1W: 48 mm × 53 mm low-signal shadow T2W: medium- and high-signal shadow Arterial phase: significantly enhanced, obvious patchy enhancement at the edge of the lesions Venous phase: some reduced, and showed uneven enhancement after delay	ALK, Desmin, vimentin	WBC, platelet, and CRP levels increased	Upper respiratory tract infection	Hepatectomy	No recurrence 21 months later
17	Costa et al. (43)	3 years/male	Jaundice, choluria, fever, abdominal pain, hepatomegaly	NM	SMA	Mild anemia Transferase, prothrombin time, and INR levels increased	NM	Liver transplantation	No recurrence 3 years later
18	Shen et al. (44)	41 years/female	Fever	MRI: an abnormal signal	Vimentin, EMA	WBC count increased	NM	Hepatectomy	No recurrence 18 months later
19	Huang et al. (45)	45 years/female	NM	A solid, well-defined mass in the right upper quadrant of the abdomen CT: a low-density mass measuring 11.2 × 8.5 × 10.5 cm, with a lower-density mass measuring 8.5 × 6.1 × 5.9 cm in the interior of the tumor	Vimentin	NM	NM	Hepatectomy	No recurrence 3 years later
20	Tong et al. (46)	65 years/female	NM	CT: a vascular enhancement mass	ALK	NM	Hysteromyomectomy	Hepatectomy	NM

CT, computed tomography; MRI, magnetic resonance imaging; NM, not mentioned; Hb, hemoglobin; T1W, T1-weighted; T2W, T2-weighted; ALL, acute lymphocytic leukemia; EMA, epithelial cell membrane antigen; ESR, erythrocyte sedimentation rate; WBC, white blood cells; SMA, smooth muscle actin; GB, gall bladder; CRP, C-reactive protein; INR, international normalized ratio; ALK, anaplastic lymphoma kinase.

Surgical resection is the most common and effective treatment for localized parenchymal tumors. In local recurrence cases, a secondary surgical resection may be employed (1). In patients with advanced-stage IMT or distant metastasis, systemic pharmacotherapy might be considered if surgical resection is not feasible (24). Currently, there is no standardized chemotherapy regimen for these cases. The overall response rate (ORR) of methotrexate-vincristine (MTX-V) chemotherapy is approximately 50% (25). Despite the limited evidence regarding the efficacy of chemotherapy in IMT, there are a few studies that suggest that chemotherapy might be useful in certain postoperative scenarios where complete tumor resection is challenging. Hence, ALK tyrosine kinase inhibitors (TKIs), such as crizotinib, ceritinib, alectinib, brigatinib, lorlatinib, and ensartinib, are beneficial for ALK-positive patients (26). In patients with metastatic or recurrent IMTs accompanied by ALK negativity, programmed death 1 (PD-1) and programmed death ligand-1 (PD-L1) inhibitors might exhibit efficacy, as PD-L1 level was enhanced in 80% of such patients (27, 28).

The recurrence of IMT depends on the tumor location. High recurrence rates are seen in sporadic or severe invasive IMTs or those with incomplete resections. Moreover, the 5-year event-free survival (EFS) and overall survival (OS) rates of IMT were 82.9 and 98.1%, respectively (2, 29). Thus, genetic testing should be performed to clarify the type of gene rearrangement to determine the prognosis. The recurrence rates of extrapulmonary and pulmonary-related IMTs were approximately 25 and 2% (30), respectively, with a metastatic rate of 5% (1). Nevertheless, the recurrence might also be associated with the tumor’s unique anatomic location or histological heterogeneity, which could cause incomplete resection. To date, the recurrence of isolated tumors post complete resection is rare (2). Since it is a malignant tumor, regular follow-up is recommended. This approach can ensure timely treatment in cases of recurrence, thereby improving the survival rate.

In this case, the patient had a history of bone marrow transplantation. The immunosuppressive state might have triggered an inflammatory disease. Since this condition can cause a delay in determining the correct diagnosis and treatment, IMT should be considered in patients with a history of immunosuppression, especially when anti-infective therapies prove ineffective. In addition, a solitary hepatic tumor was identified in this case. Despite diaphragm invasion, we achieved a complete surgical resection. During the postoperative follow-up period, no recurrence was observed. Therefore, patients can benefit from radical surgical resection in cases of localized IMTs. Immunohistochemical analysis revealed that the tumor was ALK-positive. Histological examination revealed that, the tumor comprised spindle cells. These indicators suggest a favorable prognosis.

## Conclusion

Hepatic IMT is rare. The rearrangement of the ALK gene might initiate the pathogenesis of IMT. Considering the possibility of IMT, laboratory tests and imaging studies should be analyzed to exclude other diseases. The pathological immunohistochemical examination is the preferred diagnostic criterion. Although the degree of malignancy is generally low, surgical resection is recommended for isolated IMTs due to their potential malignant tendencies. If surgery is unsuccessful in such cases, specific systemic therapies can be selected based on the various gene phenotypes. Additionally, clarifying the types of gene rearrangements may help determine the prognosis. Regular outpatient follow-up is crucial to administer effective and timely treatment in cases of tumor recurrence.

## Data Availability

The original contributions presented in the study are included in the article/Supplementary material, further inquiries can be directed to the corresponding author.
